# Drug Therapies Affecting Renal Function: An Overview

**DOI:** 10.7759/cureus.19924

**Published:** 2021-11-26

**Authors:** Reem Y Alhassani, Reem M Bagadood, Rafal N Balubaid, Haneen I Barno, Mariah O Alahmadi, Nahla A Ayoub

**Affiliations:** 1 College of Medicine, Umm Al-Qura University, Makkah, SAU; 2 Pharmacology and Toxicology, Faculty of Medicine, Umm Al-Qura University, Makkah, SAU

**Keywords:** drugs, adverse effect, renal failure, renal impairment, nephrotoxic

## Abstract

Undesirable side effects of medication are inevitable. Due to the role of the kidneys in clearance and filtration, the renal system faces a unique situation when it comes to the side effects of drugs. It has an important role for different classes of drugs to be excreted, and drugs are a key factor for this system to be at risk. Medications in articles were divided into classes using the standard set by the *Saudi Pharmaceutical Journal*. Many drug classes cause renal insults. The top six classes were pain killers, antibiotics, proton pump inhibitors, antidiabetics, antihyperlipidemics, and agents for erectile dysfunction. Renal insults caused by these agents could vary in severity. Some drugs could cause nephrotoxicity from one dose, while others may only need continuous monitoring. Different populations also operate under different rules, as some people need dose adjustments while others who are medically free of major illnesses do not. A variety of unfavorable outcomes for the kidney could take place, such as acute kidney injury, chronic kidney disease, and end-stage renal disease, and unfortunately, some of these issues could lead to the need for renal replacement therapies. The outcome of this review paper will help multidisciplinary physicians to understand the renal side effects of the most used drug classes in the Kingdom of Saudi Arabia, their destructive mechanisms, and most importantly, the clinical presentations of renal dysfunction in relation to each class. Emphasizing these adverse effects will prevent future unfavorable outcomes, especially in commonly used drugs that are frequently prescribed for different age groups. Moreover, some of these drugs are considered to be over-the-counter medications, which makes them a serious problem that needs to be handled cautiously.

## Introduction and background

Medications often cause side effects, adverse reactions, and drug-to-drug reactions. For years, physicians and patients have been concerned about undesirable side effects, which impose a significant financial burden on the healthcare system [1]. There is a wide range of side effects due to the multiple mechanisms of injury that drugs can have on the kidney. These side effects vary in severity [2]. Specifically, the incidence of drug-induced acute kidney injury (AKI) is 60% [3]. These negative outcomes sometimes force patients to undergo renal replacement therapy as a consequence [3]. It is important to note that not all people exposed to different possible nephrotoxins will develop kidney disease. Nephrotoxicity from pharmaceuticals, chemicals, and other ingested substances is the result of a complex process that includes several components, all of which must be considered. Among them are the inherent nephrotoxic potential of medications, underlying patient features that increase the likelihood of kidney damage, and the metabolism and excretion of possible offending chemicals by the kidney [4]. To effectively treat renal dysfunction caused by the use of regularly prescribed medications, it is necessary to understand the mechanisms involved and the clinical manifestations of the condition [5]. This study reviews the most prominent therapies and their adverse effects on renal function [6]. The medications studied were chosen based on the most used drug classes in the Kingdom of Saudi Arabia, as found in a literature review of articles published from 2010 to 2015 by the *Saudi Pharmaceutical Journal*. In order, the drug classes studied in this review are pain killers, antibiotics, proton pump inhibitors (PPIs), antidiabetics, antihyperlipidemics, and agents for erectile dysfunction.

## Review

Painkillers

Nonsteroidal Anti-inflammatory Drugs

Nonsteroidal anti-inflammatory drugs (NSAIDs) are one of the most common over-the-counter medications; they are analgesic, antipyretic, and anti-inflammatory. They inhibit prostaglandin synthesis by blocking the cyclooxygenase (COX)-1 and COX-2 isoenzymes, which results in the suppression of inflammation and pain. NSAIDs are used to treat various acute and chronic pain and inflammatory conditions, such as fever, dysmenorrhea, dental pain, rheumatological inflammatory diseases, and muscular pain.

In the kidneys, COX-1 regulates the renal hemodynamics and glomerular filtration rate (GFR). In comparison, COX-2 affects salt and water excretion [7,8]. NSAIDs may lead to acute interstitial nephritis secondary to allergic hypersensitivity reactions after a few days of initiating the treatment. Once intake of the drug is ceased, renal function will recover. If not, prednisone can be the solution. Long-term NSAIDs usage may result in chronic interstitial nephritis with interstitial fibrosis and chronic renal impairment. Unfortunately, groups of patients with concomitant chronic conditions are at a higher risk of developing side effects from NSAIDs than healthy individuals [9]. For patients with chronic kidney disease (CKD), liver cirrhosis, or congestive heart failure, NSAIDs should be avoided [10]. Another point must be remembered that there are some drugs, such as angiotensin-converting enzyme (ACE) inhibitors, angiotensin II-receptor blockers, and β-blockers, which may enhance NSAID-related renal complications when interacting with NSAIDs [11].

Further, a study found that in elderly patients with impaired renal function, the COX-2 inhibitor may result in a decrease in GFR, urinary sodium excretion, urinary prostaglandin E2 (PGE2), and 6-keto prostaglandin F1α excretion [12,13]. Another study showed that the kidney functions of patients with liver cirrhosis depend mainly on prostaglandin [14]. Research has shown that in 28 nonazotemic patients with cirrhosis and ascites, short-term treatment with naproxen (500 mg every 12 hours for a total of five doses) without administration of celecoxib (200 mg every 12 hours for a total of five doses) caused a significant decrease in GFR, renal plasma flow, urinary PGE2 excretion, and the suppression of diuretic and natriuretic responses to furosemide. Therefore, it is estimated that selective COX-2 inhibitors may be safer than non-selective NSAIDs in this patient population. Further information is needed to check the safety of the long-term use of selective COX-2 inhibitors in cirrhotic patients [14].

One purpose of this study was to assess whether the administration of NSAIDs has an effect on the renal function of patients with diabetes. This study hypothesized that the usage of COX-2 inhibitors in people with type 1 diabetes mellitus differs according to GFR in each case. The drug can increase or decrease GFR between individuals with euglycemia (hyperfiltration vs. normofiltration) and individuals with hyperglycemia (hyperfiltration vs. normofiltration) [15].

Opioids/Narcotics

Opioids are a class of drugs used to reduce moderate to severe pain and act as anesthesia. They are classified into natural compounds like morphine, codeine, papaverine, and thebaine. Semisynthetic compounds include diamorphine (heroin), buprenorphine, and oxycodone. Synthetic compounds include fentanyl, methadone, sufentanil, and remifentanil, and they work by binding to three types of central receptors: μ mu, κ kappa, and δ delta. Mu receptor activation causes analgesia, sedation, respiratory depression, bradycardia, and nausea. Kappa receptors produce spinal analgesia, diuresis, and dysphoria. Delta receptor activation results in spinal and supraspinal analgesia [16].

Opioids have different effects on kidney function due to the presence of opioid receptors [17]. However, their mechanisms of action are still unclear. Previous research had established that opioids protect the kidney, and this effect can be antagonized by naloxone [18].

Opioids are one of the most widely used groups of analgesics in patients with CKD [19]. As is known, the long-term administration of any drug is different from a single dose or short-term administration. Long-term opioid use in patients with CKD may lead to albuminuria, and renal parameters could change, resulting in renal dysfunction [20]. Another complication that may occur secondary to long-term opioid use is the increase in the risk of mortality and graft loss in the post-renal transplantation population [21].

Antihyperlipidemics

Statins

3-hydroxy-3-methyl-glutaryl-coenzyme A (HMG-CoA) reductase inhibitors (statins) are a group of medications used to treat high cholesterol. They work by reducing cholesterol biosynthesis in the liver, where they are distributed, and by modulating lipid metabolism derived from their effect of inhibiting HMG‐CoA reductase. There are several real benefits of statins, and these benefits outweigh the drugs' risks. Statins have antiatherosclerosis effects that cause a decrease in low-density lipoprotein (LDL) cholesterol. In addition, they can exert antiatherosclerosis effects independently of their hypolipidemic action. Additionally, statins can reduce coronary events and work as primary and secondary prevention from coronary disease, as they are the most efficient hypolipidemic compounds that have demonstrated reduced mortality rates in coronary patients [22].

The effects of statin on renal function have shown that higher-dose statin use is considered a contributing factor to AKI in older people. Using high- and medium-intensity statin regimens have been associated with significant increases in AKI hospitalization. High-intensity statin use includes 10 mg rosuvastatin, 20 mg atorvastatin, and 80 mg simvastatin or even higher doses. Medium-intensity statin therapy includes less than 10 mg rosuvastatin, 20 mg atorvastatin, 40 mg lovastatin, 80 mg fluvastatin, or 20-40 mg simvastatin. Therefore, the dose plays a significant role in increasing the risk of hospitalization with AKI for statin users [23]. The risk of AKI in patients on a statin is unknown [24].

Another study showed that statin use is not associated with AKI risk in overall populations, Caucasians, Asians, and patients undergoing cardiac and elective surgery. Statin use decreases the risk of contrast-induced nephropathy (CIN) and may increase the risk of AKI in community-acquired pneumonia patients [25].

Although the effects of statins on CKD progression are unclear, this study found that statins effectively delay CKD progression in CKD stage 3B-5 patients, particularly among those with proteinuria ≥1,000 mg/day. Therefore, statin therapy may have a net clinical benefit for preventing CKD progression, particularly considering the high dialysis burden. The protective effect of CKD on kidneys may differ according to statin dosage, and additional evidence is required to confirm these benefits [26].

Furthermore, baseline CKD status was assessed in most patients in this study, but it did not appear to influence the results. It is worth noting that a low estimated glomerular filtration rate (eGFR) does not affect blood statin levels significantly, since these medications primarily undergo hepatic metabolism rather than renal excretion [22].

Another meta-analysis evaluated the impact of statins on renal outcomes in patients with CKD. Therefore, there was a difference in eGFR. High-intensity statins were found to improve a decline in eGFR in the population with CKD, but moderate- and low-intensity statins were not. Statins were not found to decrease proteinuria in patients with CKD [27]. In CKD patients, statins like atorvastatin and fluvastatin do not require dose adjustments and are the drug of choice in patients with severe renal damage, while other statins need dose adjustments in patients with severe kidney disease (creatinine clearance [CrCl] less than 30 mL/min) [28,29]. Regarding fatal consequences, one of the most severe adverse effects is myotoxicity, resulting in rhabdomyolysis that may cause acute renal failure, disseminated intravascular coagulation, and death [30].

Fibrates

Fibric acid derivatives (fibrates) are a class of medication that lowers the level of triglycerides. It is used as a second-line agent behind statins. It decreases triglyceride levels by reducing the liver's very-low-density lipoprotein (VLDL) production and removing triglycerides from the blood. Fibrates are also effective in increasing blood high-density lipoprotein (HDL) cholesterol levels; however, fibrates are not effective in lowering LDL cholesterol. They also tend to reduce LDL cholesterol levels and increase HDL cholesterol [31]. Fibrates also work by activating a protein called peroxisome proliferator-activated receptor alpha (PPAR-alpha). PPAR-alpha activates the enzyme lipoprotein lipase, resulting in the decreased formation of VLDL cholesterol (which is converted into LDL cholesterol) triglycerides and an increase in HDL cholesterol [31].

The adverse-effect profile of fibrates is related to renal function by a reversible increase in serum creatinine values. The first explanation is that fibrates increase creatinine production, in which case an increase in serum creatinine values would not represent an actual change in renal function. An alternative explanation is that fibrates reduce vasodilatory prostaglandin production, which would lead to a real difference in renal function, causing an increase in creatinine levels [32,33].

Regular renal function monitoring should be considered essential during fibrate administration, particularly in preexisting renal disease. A 30% increase in serum creatinine values could be due to fibrate use, so discontinuing the use of the drug is necessary. After discontinuation of a fibrate, serum creatinine values should be administered for several weeks to return to baseline [32,33].

Proton pump inhibitors

PPIs are one of the most common over-the-counter drugs taken by millions of patients worldwide. Their use periods could be quite long and range from months to years [34,35]. Studies have found that >50% of prescribed PPIs are unnecessary, especially for the elderly [36,37].

This drug group inhibits the hydrogen potassium adenosine triphosphatase (H+/K+-ATPase) enzyme and stops the exchange of K+ and H+, increasing gastric acid pH and blocking its secretion [38]. Clinical uses for PPIs include gastroesophageal reflux disease, peptic ulcers, acid-related dysphagia, and erosive esophagitis. It is also used as a prophylactic in NSAID users to decrease peptic ulcer risk [35,39]. It has been proven that PPIs have several adverse effects on renal function, and most studies found an association between acute interstitial nephritis (AIN) and PPI use [40].

A systematic review and meta-analysis summarized these effects into four significant outcomes: AIN, AKI, CKD, and end-stage renal disease (ESRD) [41]. The first recognized association between AIN and PPIs was made in 1992. Nowadays, PPIs are considered one of the most common causes of drug-induced AIN [34]. A prospective cohort study found that PPI consumption is an independent risk factor for CKD and AKI [42]. Interestingly, a retrospective case-control study stated that younger patients are more likely to develop CKD. Another cohort study said it is more common for older patients to develop AKI and AIN associated with PPI use [35,43]. CKD patients who need to be treated with PPIs should be under creatinine level monitoring.

However, there is no need for dosage adjustments in patients with underlying kidney disease. Despite the numerous studies on this topic, all conclusions have been made observationally, which might have caused the results to be subject to significant bias. However, acute insult is treated by the withdrawal of PPIs, corticosteroid administration, and renal replacement therapy if needed [34,38]. Physicians should be aware of the associated adverse reactions to the kidney. However, other obvious precipitating factors should be excluded before the discontinuation of PPIs [40].

Antidiabetics

Metformin

Globally, metformin is recognized as the first line of management for type 2 diabetes [44-46]. Regardless of the antihyperglycemic effects of this drug, there is another advantage, such as preventing the risk of hypoglycemia and weight gain, decreasing the risk of macrovascular complications (sudden death, stroke, acute coronary syndrome, and peripheral vascular disease), improving insulin resistance and lipid profiles, and having a low cost [45-47]. This medication is considered biguanide, which decreases hepatic gluconeogenesis by shifting metabolism from aerobic to anaerobic, and can reduce adipocytes, muscle, and liver lipogenesis [48]. Because it is classified as a biguanide, there is a belief that lactic acidosis is a side effect of metformin. Lactic acidosis is a serious but rare condition; recently, there has been no evidence found between metformin usage and elevated risk of lactic acidosis. Some papers have correlated this condition to the disease itself, not the therapy. Therefore, metformin can easily be eliminated through the urine but can accumulate in kidney impairment patients [49-51]. The Food and Drug Administration (FDA) has a new recommendation to use metformin depending on the eGFR rather than the serum creatinine level.

If the eGFR is ≥60 mL/min/1.73 meter per square (m^2^) or >45 to <60 mL/min/1.73 m^2^, there is no need for dose adjustment; instead, renal function should be monitored at least once annually. If eGFR is 30-45 mL/minute/1.73 m^2^, the initiation of therapies is not recommended while the continuation of existing therapy may occur at a reduced dose up to a maximum of 500 mg twice daily with close monitoring of kidney function. Finally, if eGFR is <30 mL/min/1.73 m^2^, it is contraindicated to use drugs. In AKI, the patient should be instructed to temporarily hold on metformin administration [52,53]. This result decreases the usage of metformin among the population [54-57].

Sulfonylureas

Sulfonylurea is the oldest antidiabetic therapy available worldwide [58]. This drug is low in cost and plays a vital role in controlling glycemic levels; therefore, it is the second-line management depending on the guidelines, such as the National Institute for Health and Care Excellence (NICE), the American Diabetes Association (ADA), and the European Association for the Study of Diabetes [59]. Sulfonylurea improves hyperglycemia levels by acting on pancreatic B-cells to secrete insulin and decrease insulin resistance [60]. Due to this mechanism of action, there is a high risk of hypoglycemia. Additionally, weight gain has been recognized as a side effect of this medication [60,61]. In patients with renal dysfunction, the risk of hypoglycemia is high because active metabolites accumulate and are not excreted by the kidney. When we use drugs from the sulfonylurea class, especially glibenclamide and glimepiride, we should reduce the dose in cases of renal impairment and contraindications in stage ≥3 CKD (eGFR < 60 mL/min). When using gliclazide and glipizide, the liver can convert metabolites from active to inactive. This makes it easy to eliminate these substances through urine, so this leads to fewer hypoglycemia events and no need to reduce dose in stages 1-3 CKD. An adjusted gliclazide dose is permitted in severe CKD, but the dose of glipizide does not need to be reduced to be safe, although there is still the need for caution against hypoglycemia risk [62,63].

Insulin

The liver degrades endogenous insulin; in contrast, exogenous insulin is eliminated by the renal system. A well-known side defect of insulin is hypoglycemia. The dose should be reduced by 25% if eGFR is 10-50 mL/min, and when eGFR is less than 10 mL/min, the dose should be reduced by 50% [64].

GLP-1

Glucagon-like peptide-1 (GLP-1) receptor agonist is an injectable drug that can treat type 2 diabetic patients by acting GLP-1 receptors and increasing the secretion of glucose-dependent insulin in contrast to reducing glucagon [65,66]. Another advantage is that increased satiety by slowing the emptying of the gastric system leads to weight loss and improves cardiorenal risk factors involving lipid levels and blood pressure in type 2 diabetic patients. Obesity, hypertension, and hyperglycemia contribute to the development of renal and heart diseases, so this medication can decrease the progression or onset of CKD [67-70]. The most common disadvantage is a gastrointestinal side effect (diarrhea or vomiting) leading to dehydration, which can affect renal function. In stage 3 CKD, we need to closely monitor and reduce the dose of exenatide to 5 μg and not allow it to be used in stages 4 or 5. Liraglutide is not eliminated in feces or urine but is degraded in the body. Therefore, we can use it in all CKD stages. Current studies have not indicated positive results regarding liraglutide’s effectiveness and safety in patients with stage 3 or higher CKD [26,27].

DPP-4

Dipeptidyl peptidase-4 (DPP-4) inhibitors used to treat type 2 diabetes are considered a new oral medication of antidiabetic therapy. This drug inhibits the DPP-4 enzyme, which degrades GLP-1 and gastric inhibitory polypeptide (GIP) hormones that are secreted following a meal and act to secrete insulin [71]. In renal impairment, dose adjustment is recommended because all agents in this drug category, except linagliptin, are eliminated by the kidney [72].

Antibiotics

Aminoglycosides

Aminoglycosides are protein inhibitor antibiotics used in empirical therapies for severe infections, such as those arising from septicemic, respiratory, urinary, and complicated intra-abdominal infections caused by aerobic gram-negative bacilli [71,72]. One of the most severe side effects of aminoglycosides is nephrotoxicity. Additionally, it can cause renal tubular dysfunction and an increase in serum creatinine [73]. The highest rates of nephrotoxicity were reported in gentamicin [74]. A meta-analysis revealed no statistical difference between the incidences of nephrotoxicity among patients who received single daily doses rather than multiple daily doses of aminoglycosides [75]. A case report indicated gentamicin acute tubular necrosis after a single-dose exposure [76]. The molecular mechanisms of toxicity were not precise. Histopathological investigations have promoted the concept of tubular necrosis at the apical membrane [77]. The first site of aminoglycoside-cell interaction occurs in the renal proximal tubular cells, which selectively transport and accumulate drugs, leading to the transportation of abnormalities to the brush border and basolateral membranes [78]. Tubular regeneration and kidney function recovery are expected after 20 days following the cessation of the medication [79].

Fluoroquinolones

Fluoroquinolones are direct inhibitors of DNA synthesis and are antibiotics used to treat atypical and hospital-acquired respiratory tract infections. Fluoroquinolones include ciprofloxacin, levofloxacin, and norfloxacin. Ciprofloxacin contributes to nephrotoxicity [80]. The most common nephrotoxicity is allergic interstitial nephritis, a type III hypersensitivity reaction that clinically presents with fever, skin rashes, acute arthralgia, eosinophilia, proteinuria, eosinophiluria, hematuria, and pyuria [81]. Another side effect is acute renal failure due to a hypersensitivity reaction. Most cases resolve within one week to two months following discontinuation of the drug [82]. Other side effects involve granulomatous interstitial nephritis, crystalluria, and acute tubular necrosis [83]. Long-term outcomes are reassuring, with a return to baseline renal function in less than a month of termination [84].

Vancomycin

Vancomycin is a cell wall synthesis inhibitor that inhibits bacterial growth and works through its affinity to bind to the terminus part of the peptidoglycans in the bacterial cell walls [85]. This results in inhibiting bacterial cell wall biosynthesis [86].

Vancomycin is used against gram-positive multidrug-resistant microorganisms, and it is the first choice in treating infections with methicillin-resistant *Staphylococcus aureus* (MRSA) [85,86].

Given the increased usage of vancomycin to deal with drug-resistant bacteria over the past several years, vancomycin-induced nephrotoxicity (VIN) has become an important issue associated with multiple dilemmas. One dilemma arises from the inaccurate definition of VIN, leading to a common delay in its diagnosis; therefore, it has been highlighted that sufficient criteria should recognize the definition of VIN [87].

High dosage is the main factor, as the plasma trough level of vancomycin plays a significant correlation with nephrotoxicity [88], especially doses of >20 mg/L or >4 g/day, and it has a linear relationship with the incidence of AKI [89]. Furthermore, a high dose of vancomycin has been found to be an independent risk factor for nephrotoxicity if the dose is ≥15 mg/L [90].

Another two factors for VIN are the prolonged duration (more than seven days) and synergism with other nephrotoxic agents [91]. As an emphasis on the duration factor, many studies have agreed on the definition of exceeding a seven-day period [89,90,92], but it was shown in a study that the risk factor of the long period is not harmful when the dose is low (<15 mg/L) [93]. The use of a combination of vancomycin with some other antibiotics to have a broader spectrum of effect is a further essential factor that causes VIN.

One of the highlighted combinations is synergism with piperacillin and tazobactam [94] and a combination with an aminoglycoside [95]. A study indicated the importance of careful use of an empirical combination of antimicrobial therapy with vancomycin [96]. Another reasonable factor for VIN is impaired glomerular filtration. This impairment is either caused by an existing renal disease like CKD [96] or by clinical events that compromise renal function directly or indirectly by causing hemodynamic instability like hypotension [89].

Regarding pathophysiology, biopsies taken in some case reports showed that VIN can take several forms, such as injury of the proximal tubular cell with or without necrotic changes, acute interstitial nephritis, and previous nephrotic changes in the biopsy [97].

The mechanism of action is that vancomycin produces oxidative substances like superoxide, which generates free radicals and therefore reduces the activity of antioxidative enzymes [98].

Furthermore, superoxide production by vancomycin depolarizes the mitochondrial membrane, allowing subsequent apoptotic cell death [98]. One therapeutic agent studied through a cell culturing laboratory experiment to reverse the effects of VIN is cilastatin. The observation focused on reversing the impact of VIN in renal tubules [99]. Cilastatin reduces VIN-induced proximal tubular cell damage, protects against early and late apoptosis (but not necrosis), downgrades mitochondrial damage, improves cell recovery after the use of vancomycin, and reduces the intracellular accumulation of vancomycin [100,101].

Furthermore, there are several agents tested on animals that have been found to increase the survival of renal tubular cells. They mainly work as antioxidative agents that can reduce DNA fragmentation, apoptosis, and necrosis, of which are hexamethylenediamine-conjugated superoxide dismutase, erdosteine, vitamin E, vitamin C, N-acetylcysteine, caffeic acid phenethyl ester, and recombinant human erythropoietin [100,101].

Drug-drug interactions of cited drugs are listed in Table 1. The summary of cited drugs is provided in Table 2.

**Table 1:** Drug-drug interaction between drugs classes.

Drug-drug interaction	Affect	Patient management	Reference
Aminoglycosides/nonsteroidal anti-inflammatory agents	Nonsteroidal anti-inflammatory agents may decrease the excretion of aminoglycosides.	Monitor for increased nephrotoxic effects of aminoglycosides if a nonsteroidal anti-inflammatory agent is initiated or the dose is increased. This is of particular concern in preterm infants.	[102]
Aminoglycosides/vancomycin	Vancomycin may enhance the nephrotoxic effect of aminoglycosides.	Monitor for increased nephrotoxic effects of aminoglycoside if administered concomitantly with vancomycin. Monitor aminoglycoside serum concentrations carefully and adjust dosing accordingly.	[103]
Quinolones/nonsteroidal anti-inflammatory agents	Nonsteroidal anti-inflammatory agents may enhance the neuroexcitatory and/or seizure-potentiating effect of quinolones. Nonsteroidal anti-inflammatory agents may increase the serum concentration of quinolones.	Consider the increased risk of seizure that may accompany the concomitant use of nonsteroidal anti-inflammatory agents and quinolone antibiotics. Additional factors that may be associated with an increased risk of such interaction include renal dysfunction, history of seizure or other neurological disorder, and high doses/serum concentrations of either agent.	[104]

**Table 2:** Summary of renal adverse effects for each class.

Pharmacological action	Class of the drug	Renal adverse effect
Painkiller drugs	Nonsteroidal anti-inflammatory agents	Cause chronic renal impairment if used for a long time. In acute settings, can lead to acute interstitial nephritis.
	Opioids	Multiple effects on renal function but the mechanism of function is still unknown. It is approved that long-term usage of opioids results in albuminuria and change in the renal parameter.
Antihyperlipidemic drugs	Statin	Recognized as a risk factor to acute kidney injury in older people.
	Fibrates	The adverse effect is a reversible rise in serum creatinine values.
Proton pump inhibitor drugs	Proton pump inhibitor	Cause drug-induced allergic interstitial nephritis, acute kidney injury, chronic kidney injury, and end-stage renal disease.
Antidiabetic drugs	Metformin	In patients with kidney impairment, prefer to adjust doses so it will not get accumulated.
Antibiotics drugs	Aminoglycosides	Nephrotoxicity is caused by a single dose, but kidney function is recovered 20 days after the cessation.
	Fluoroquinolones	Allergic interstitial nephritis, a type III hypersensitivity reaction. Most cases resolve within one week to two months of discontinuation.
	Tetracycline	Tetracycline is a protein synthesis inhibitor. Doxycycline has a role in decreasing proteinuria in diabetic nephropathy patients.
	Vancomycin	A cell wall synthesis inhibitor. Vancomycin-induced nephrotoxicity has been an important issue associated with multiple dilemmas.

## Conclusions

The results of this study state that these classes of drugs have many side effects on renal function. In some classes, a particular drug can be the only renal insulting agent. The results also showed how various drugs could increase renal insult when combined together, as was seen with NSAIDs and vancomycin. In different classes, the mechanisms leading to renal injury varied between clear, unclear, and a hypothesis. A single dose is enough for some drugs to be acutely nephrotoxic, which was seen in gentamicin, or nephrotoxicity can be chronic with long-term usage of some drugs, like NSAIDs. Others are sufficiently nephrotoxic when used in the long term and in a high dosage, which is the case for VIN. The definition of long-term usage for different drugs is different. For the same drug, nephrotoxicity may or may not occur for different populations, a phenomenon manifested with the antihyperlipidemic statin, which is considered a contributing factor to AKI in older people. The results showed that kidney function assessments before the administration of a nephrotoxic drug should not be limited to assessing only serum creatinine; this was proved true in metformin administration. Solutions to reverse or inhibit renal complications were also discussed. However, in some cases, the solution could only be to avoid the insulting agent for a certain population or to ensure continuous clinical monitoring in a healthcare facility, especially with the long-term use of a drug. The results of this study should encourage physicians to study a drug’s mechanism of action and its effects on kidney function in a patient before any administration of a drug that probably affects the kidney (or not) to prevent unwelcomed outcomes that can lead to a lower quality of life or increased mortality.
